# Transcriptomic Network Regulation of Rat Tooth Germ from Bell Differentiation Stage to Secretory Stage: MAPK Signaling Pathway Is Crucial to Extracellular Matrix Remodeling

**DOI:** 10.1155/2023/4038278

**Published:** 2023-02-11

**Authors:** Huiru Li, Xiaoyan Hu, Ailin Zen, Yujiong Chen, Minzhi Yang, Jian Zhang, Jing Tang, Qin Fan, Shanshan Feng, Jianguo Liu, Mingsong Wu

**Affiliations:** ^1^School of Stomatology, Zunyi Medical University, Zunyi, Guizhou, China; ^2^Special Key Laboratory of Oral Disease Research of Higher Education Institution of Guizhou Province, Zunyi Medical University, Zunyi, Guizhou, China; ^3^Department of Pathology, Affiliated Hospital of Zunyi Medical University, Zunyi, Guizhou, China

## Abstract

Hard tissues make up the vast majority of teeth and are mineralized from the surrounding matrix. If the development of tooth germ is affected during mineralization, hypoplasia of the tooth tissue can occur. To better understand the mechanisms mediating hypoplasia, we need to first study normal development. Using a rodent model, we highlight the transcriptomic changes that occur from the differentiation to secretion stages of mandibular molar germs. The tooth germ was dissected from rats at postnatal day 1.5 or 3.5 for high-throughput sequencing. Combining transcriptome analysis and DNA methylation, we identified 590 differentially expressed genes (436 upregulated and 154 downregulated) and 551 differentially expressed lncRNAs (long noncoding RNA; 369 upregulated and 182 downregulated) which were linked to the biological processes of odontogenesis, amelogenesis, tooth mineralization, and the alteration of extracellular matrix (ECM), especially matrix metalloproteinases (MMPs) and elastin. We found DNA methylation changes in 32 selected fragments involved in 5 chromosomes, 26 targets, and 2 haplotypes. Finally, three novel genes were identified: MMP20, Tgfb3, and Dusp1. Further analysis revealed that MMP20 has a role in odontogenesis and amelogenesis by influencing Slc24a4 and DSPP; Tgfb3 is involved in epithelial cell proliferation, cellular component disassembly process, ECM cellular component, and decomposition of cell components. But lncRNA expression could affect DNA methylation and mRNA expression. Moreover, the degree of DNA methylation could also affect the transcriptome level. Thus, Tgfb3 had no difference in DNA methylation, and Dusp1 conferred no difference at the transcriptome level. These three genes were all enriched in the MAPK pathway and played an important role in ECM remodeling. These data suggest that during the period of the bell differentiation stage to the secretory stage, along with enamel/dentin matrix secretion and hard tissue occurrence, the ECM is remodeled via MAPK signaling.

## 1. Introduction

Odontogenesis is a complicated and precise process which refers to mutual induction and interactive events between the oral epithelium and mesenchyme originating from the neural crest. Tooth development depends on the interaction of multiple signal molecules and location signals instead of being controlled by a single molecule or signaling. It is universally acknowledged that principal regulatory genes and pathways include sonic hedgehog (SHH) [1], fibroblast growth factors (FGF) [2], transforming growth factor-beta (TGF-beta) [3], bone morphogenesis protein (BMP) family [4], and epidermal growth factor (EGF) pathway [5]. Furthermore, Notch and wingless (Wnt) signaling pathways are also gradually recruited to make contributions to odontogenesis [6, 7]. However, due to the temporal and spatial complexity of gene expression, function, and regulation, ongoing research endeavors are aimed at figuring out how genes and their signaling mechanisms work precisely.

Traditionally, tooth germ development is divided into the bud, cap, and bell stages. Because amelogenesis and dentinogenesis begin at the bell stage, they can also be divided into three stages: morphogenetic, differentiation, and secretory [8]. During the bell differentiation stage, the mature inner enamel epithelium differentiates into elevated columnar preameloblasts at the junction of inner enamel epithelium and dental papilla, and the dental papilla cells begin to differentiate into preodontoblasts and secretory odontoblasts. At the tip of the dental papilla, some odontoblasts begin to secrete predentin. Until the secretory phase, the ameloblasts are further differentiated into mature ameloblasts with a secretory function, and the preodontoblasts are differentiated into high-column mature cells. The enamel matrix and dentin matrix are secreted, and the dentin layer begins to form. Genes influence phenotype change. Among this process, TGF-beta and BMP signaling pathways take part in cell differentiation and matrix protein production through a cross-talk between Smad and non-Smad pathways [9]. Additionally, large hyaluronate-binding proteoglycans 5D5, amelogenin Exon4, and laminin-5 subunits are involved in the differentiation and secretion of ameloblast and osteoblast [10–12]. Understanding the transition from differentiation to secretion status warrants a better understanding of the genes involved and what they alter.

Herein, we use a RNA sequencing approach to identify the underlying gene changes. Aside from DNA sequence, the gene expression pattern is also influenced by epigenetic information and environment. Even monozygotic cotwins with the same genome show different dental phenotypes [13]. This highlights that epigenetic and environmental factors also contribute to the variation. While epigenetics mainly refers to noncoding RNA modulation, histone modification, DNA methylation, and chromatin remodeling, it also includes siRNA, miRNA, gRNA, and lncRNA. With higher-resolution research, miRNAs have been implicated in tooth development [14, 15]. Indeed, miRNAs have different effects on the various stages of tooth development and may regulate the proliferation and differentiation of stem cells [16, 17]. Currently, only a few articles have investigated the role of lncRNA. Some find that lncRNA H19 promotes the differentiation of dental stem cells [18]. Others report that the lncRNA DANCR sponges miR-216a to react upon odontoblast differentiation, and Williams et al. find that colorectal cancer-associated genes (lncRNA CASC8) are linked to tooth agenesis [19, 20]. Nevertheless, there has been minimal research on how lncRNA directly impacts tooth development. Furthermore, although new technologies allow for the analysis of DNA methylation [21], neither lncRNA nor DNA methylation has been sufficiently investigated in the context of tooth development. Thus, we focus our study on the involvement of epigenetic DNA methylation and lncRNA modulation in the differentiation to secretion stages of tooth development.

## 2. Material and Methods

### 2.1. Animals

Specific pathogen-free Sprague-Dawley (SD; 8 weeks old) rats were provided by the Laboratory Animal Center of Zunyi Medical University. There were two male rats and four female rats in total. Adult rats were paired randomly, and every male rat was housed with two female rats in an animal house maintained at 25°C and fed normal commercial pellets and water ad libitum. Rats can copulate freely and give birth. Rat pups were experimentally used at postnatal days 1.5 and 3.5. All animal experiments are in compliance with the ARRIVE guidelines [22] and carried out in accordance with the U.K. Animals (Scientific Procedures) Act, 1986, and associated guidelines (EU Directive 2010/63/EU) for animal experiments. Furthermore, the experiments were reviewed and authorized by the ethics committee of the Hospital of Stomatology, Zunyi Medical University (Permit Number: YJSKTLS-2019-2021-029A).

### 2.2. Tooth Germ Preparation

**Figure 1:**
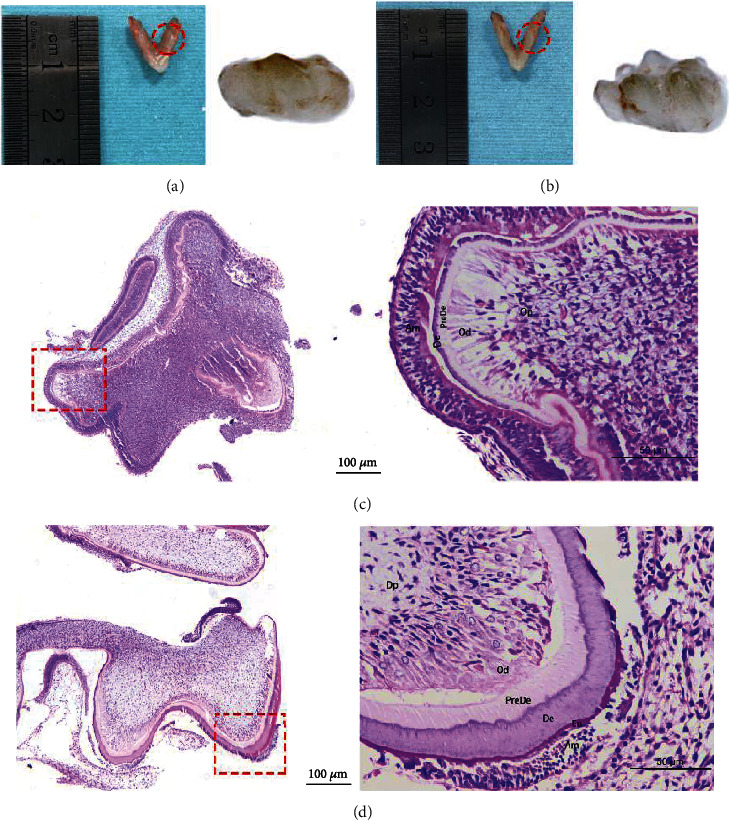
Mandibular molar germ and its histological morphology. (a) There was no obvious mineralized tissue in the P1.5 tooth germ under the microscope, and there were small apex protrusions. (b) Microscopically, tooth germ of P3.5 had obvious mineralized dental cusp tissue, and the mineralized layer was thin. (c, d) H&E staining of global and local structures of mandibular molar germ in P1.5 (up) and P3.5 (down). With the development of germ, the mineralized structure gradually widened. Am: ameloblast; En: enamel; De: dentin; PreDe: predentin; Od: odontoblast; Dp: dental papilla.

SD rats with a tooth in the differentiation stage at postnatal day 1.5 (P1.5) and secretory stage at postnatal day 3.5 (P3.5) were treated with inhalation anesthesia. We chose a 30% volume displacement rate per minute for CO_2_ euthanasia of rodents and assisted by cervical dislocation. After euthanasia, we separate the head along the upper neck and then dissected the mandible along the corner of the mouth. The location of the mandibular molar tooth embryo was found at the mandibular ascending ramus below the coracoid process. Finally, tooth germs were dissected with ophthalmic tweezers under a stereo microscope (Olympus SZ61, Tokyo, Japan) and photographed with a microscope camera (MSHOT, Guangzhou, China) (Figures 1(a) and 1(b)). Samples were put in solidified carbon dioxide and immediately sent out for high-throughput sequencing (Genesky Biotechnologies Inc, Shanghai, China). We compared gene expression from the secretory stage to the differentiation stage, and each group was prepared as three individual samples. In addition, we prepared three additional rats per group for follow-up validation experiments. To ensure their development stage, pups outside the expected size range were excluded.

### 2.3. Hematoxylin-Eosin Staining

Following molar extraction, samples were fixed with 4% paraformaldehyde for 24 hours. Tissues were embedded in paraffin after washing, dehydration, transparency, and wax immersion for section staining. Slices stained with hematoxylin and eosin (H&E) were sealed with neutral gum and photographed under a microscope (Leica, Wetzlar, Germany) (Figures 1(c) and 1(d)).

### 2.4. RNA Sequencing and DNA Methylation Assessment by MethylTarget Technology

Briefly, the molar germs were used for RNA and DNA isolation. The total RNA was isolated by the TruSeq RNA Sample Preparation Kit v2 (Illumina, USA), and the DNA was isolated using a DNA extract kit (Concert, Xiamen, China) after being treated with the bisulfate EZ DNA Methylation-Gold™ Kit (ZYMO RESEARCH, California, USA). The unmethylated cytosine C of genomic DNA was converted to uracil U. And the following procedures of purifying, fragmenting, amplifying PCR, building library, and sequencing were outsourced to Genesky (Shanghai, China). The library was sequenced on an Illumina HiSeq platform in a 2 × 150 bp paired-end sequencing mode. Our quality control data are shown in supplementary data, and the DNA methylation primers are listed in Table 1.

### 2.5. Gene Ontology Analysis and Kyoto Encyclopedia of Genes and Genomes Analysis

The identification of differentially expressed genes of mRNA (DEGs) and lncRNA (DE-lncRNA) was performed using the clusterProfiler package. Genes with *p* values < 0.05 and |logFC| > 1 were taken as standard. Based on those DEGs and DE-lncRNA, genes were enriched in the gene ontology (GO) and Kyoto Encyclopedia of Genes and Genomes (KEGG) pathways.

### 2.6. Protein-Protein Interaction (PPI) Network Analysis

**Figure 2:**
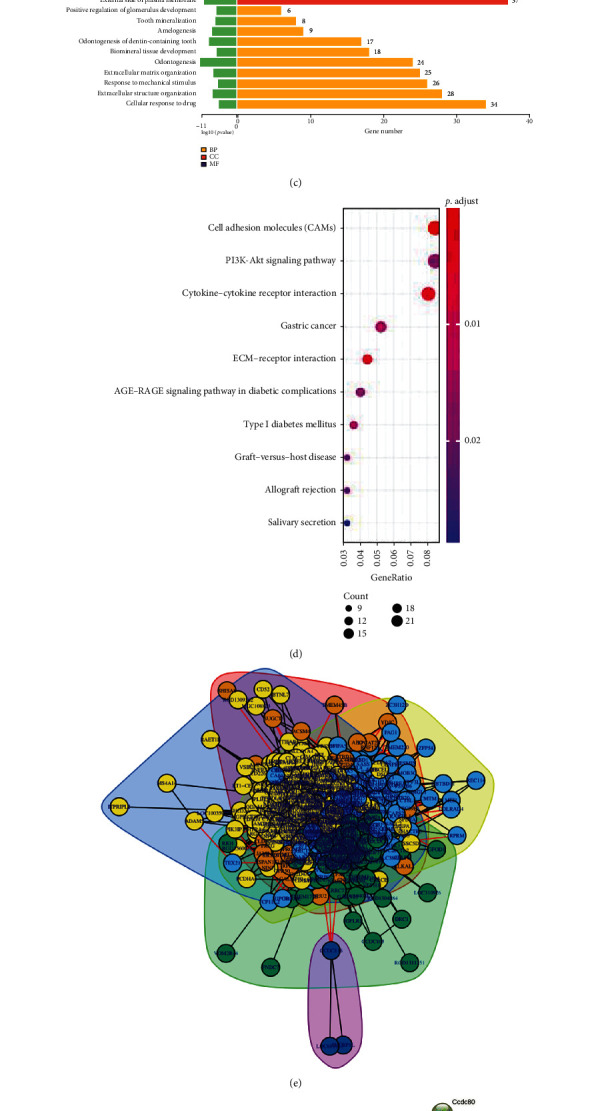
mRNA differential expression analysis. (a) DEGs clustering heatmap (top 50). Each column represents different samples, and the lines represent different genes. Red indicates that the gene was highly expressed in the sample, and blue indicates that the gene was lowly expressed in the sample. As the difference is greater, the color is deeper. (b) Red dots represented upregulated genes, purple dots represented downregulated genes, and gray dots represented genes with no significant change. The *Y*-axis was -log10 (*p* value), and the bigger quantity reflects a lesser difference (only the data with a *p* value < 0.05 are shown). (c) Every ten most significantly enriched GO terms were selected from the three categories of BP (purple), CC (orange), and MF (yellow), displayed in the figure. If there were less than 10 GO terms, they were all displayed. (d) *X*-axis is gene ratio, indicating the ratio of differentially expressed mRNAs under the pathway entry to the total number of DEGs. The larger gene ratio value reflects a higher enrichment of DEGs in the KEGG pathway. The bubble size indicates the number of enriched DEGs, and the color represents the *p* value magnitude. (e) Clustering results of differentially expressed genes. (f) PPI network of the biggest clusters (minimum required interaction score is high confidence): only the included proteins were found to correspond to other proteins.

According to a *p* value < 0.05 cut-off and variational expression, DEGs were used to base on predicting protein interactions by STRING which is a database of known and predicted protein interactions, including direct physical interactions and indirect functional correlations. Furthermore, this network was clustered by the fast greedy algorithm function in the igraph R package (Figure 2(e)). After clustering, GO and KEGG enrichment analyses were performed for modules with more than 10 differentially expressed genes.

### 2.7. Real-Time PCR

Total RNA was isolated with the TRIzol® Reagent (Invitrogen™ Life Technologies, Carlsbad, USA) according to the manufacturer's instruction. First-strand synthesis was performed by the RevertAid First Strand cDNA Synthesis Kit (Thermo Scientific™, Massachusetts, USA), followed by real-time PCR (RT-qPCR) using a DyNAmo Flash SYBR Green qPCR Kit (Thermo Scientific™). Primers were designed for transforming growth factor-beta 3 (*Tgfb3*), matrix metalloproteinase 20 (*MMP20*), and dual-specificity phosphatase 1 (*Dusp1*) as shown in Table 2.

### 2.8. Statistical Analysis

All experiments were performed with at least three biological replicates. Statistical analysis was performed using SPSS17.0 software. The normal distribution data were expressed as the mean ± standard deviation ($\bar{x}\pm s$), and the skewness distribution data were expressed as the median (quartile spacing). The *t-*test was used for comparisons between the two groups. For the identification of DMC and DMR, Bi Seq (1.6.0) and a methyl kit (1.0.0) were used. In addition, *p* < 0.05 was the threshold for statistical significance.

### 2.9. Mapping Software

All figures were constructed by TBtools [23], the R programming language, and Draw Venn Diagram. TBtools was used to draw heatmaps. The R programming language was used to map the volcano plot, scatter diagram, cluster map boxplot, and network diagram. Draw Venn Diagram was used to draw Venn diagrams, and the histogram of mRNA expression was drawn using GraphPad Prism 9.0.

## 3. Results

### 3.1. RNA Sequence Analyses

To investigate changes in the genetic regulatory network in tooth germ development, we analyzed DEGs using RNA sequencing. There were 590 DEGs in the mRNA sequence (436 upregulated and 154 downregulated) (Figures 2(a) and 2(b)). Furthermore, based on these DEGs, among the 1,129 GO pathways significantly enriched in mRNA sequence, there were 921 terms referred to biological process (BP), 121 to cellular component (CC), and 83 to molecular function (MF) (Figure 2(c)). In terms of the KEGG pathway, there were 261 pathways significantly enriched in mRNA of which 48 pathways were notable (Figure 2(d)). In the GO enrichment items, the DEGs were linked to the biological process of odontogenesis and amelogenesis, tooth mineralization, and the alteration of the extracellular matrix (ECM). ECM remodeling processes were affected by matrix metalloproteinases (MMPs) which can degrade collagen and elastin and influence Ca^2+^ signaling [24]. As a family of zinc-dependent endopeptidases, MMPs, especially MMP20, have shown to hydrolyze dentin sialophosphoprotein (DSPP) into dentin phosphoprotein (DPP), dentin sialoprotein (DSP), and dentin glycoprotein (DGP) during odontogenesis and are involved in amelogenesis [25]. Indeed, *MMP20* was upregulated, indicating that the metal iron transmembrane activity was so active that the change rebuilt ECM, ECM structural constituent, ECM component, ECM organization, collagen trimer, ion channel activity, and odontogenesis of the dentin-containing tooth. Moreover, MMPs can be induced by the extracellular matrix metalloproteinase inducer (EMMPRIN, also named CD147), which is located in the membrane raft, by the mitogen-activated protein kinase (MAPK) p38 pathway [26].

During the process of tooth development, ion channel dynamics are altered including changes in chloride channels, calcium channels, potassium channels, transient receptor potential vanilloids, and solute carrier superfamily members [27]. In our study, we found that sodium ion transmembrane transporter activity changed and activated sodium channels. This channel is believed to modulate channel kinetics and cell adhesion due to its *β* subunits which act as a kind of adhesion molecule that interacts with ECM proteins [28]. Thus, there was significant enrichment in the cell adhesion molecule (CAMs) pathway (rno04514). The activation may be caused by ameloblasts. There are two biological processes related to ameloblasts which have been shown to activate sodium channels. When ameloblasts synthesize and absorb enamel matrix proteins, Ca^2+^ needs to be transported and extruded, at the same time Na^+^ is exchanged by Na^+^-Ca^2+^ exchanger (NCX) which belongs to the solute carrier (SLCs) superfamily *Slc8a* [29]. The other one is shown in the period of ameloblast differentiation and enamel matrix formation. Ameloblast strictly controls glucose metabolism through a sodium-dependent active glucose transporter that is encoded by *Slc5a* [30]. In our results, *Slc5a5* was upregulated while *Slc13a3* was downregulated. Additionally, *Slc4a5*, *Slc4a8*, *Slc6a2*, *Slc6a15*, *Slc13a5*, *Slc22a3*, and *Slc24a4* were upregulated. Among these genes, *Slc24a4* was coenriched in metal ion transmembrane transporter activity (GO:0046873), odontogenesis of dentin-containing tooth (GO:0042475), odontogenesis (GO:0042476), and amelogenesis (GO:0097186) with *MMP20*. Furthermore, *Dmp1* and *Dspp* also contributed to amelogenesis and odontogenesis.

The PPI network is a tool that can investigate protein interaction, cellular function, and disease mechanisms. In the study, we constructed a PPI network by mapping the DEGs into PPI data in STRING and found 584 nodes and 1,392 edges in the network. And then, we clustered the network by the fast greedy algorithm and then finished GO and KEGG pathway analysis of modules with more than 10 differentially expressed genes at the same time to identify network clusters (Figure 2(f)). Finally, there were 12 clusters. The biggest cluster enriched 147 proteins which included MMP20, Cd4, Fgf4, and Tgfb3. Among those proteins, MMP20 interacted with Ambn, DSPP, Dmp1, and Itgb3. Apart from Itgb3, the encoding genes of those proteins were enriched regulatory genes of odontogenesis of dentin-containing, while Tgfb3 was relative to Tgfbr2 and Itgb3. Thus, MMP20 had an indirect relationship with Tgfb3 through Itgb3. We found that both MMP20 and Tgfb3 took part in cellular component disassembly and ECM cellular component. Therefore, in general, during the transformation from the bell differentiation stage to the secretory stage, through the MAPK signaling pathway, the CD147 gene in ameloblasts affected the expression of MMP20, an ECM remodeling-related gene. Concurrently, Tgfb3 was involved in the decomposition of cell components and epithelial cell proliferation. Therefore, in the process of development, genes related to ECM underwent vast changes.

### 3.2. lncRNA and mRNA Coexpression

**Figure 3:**
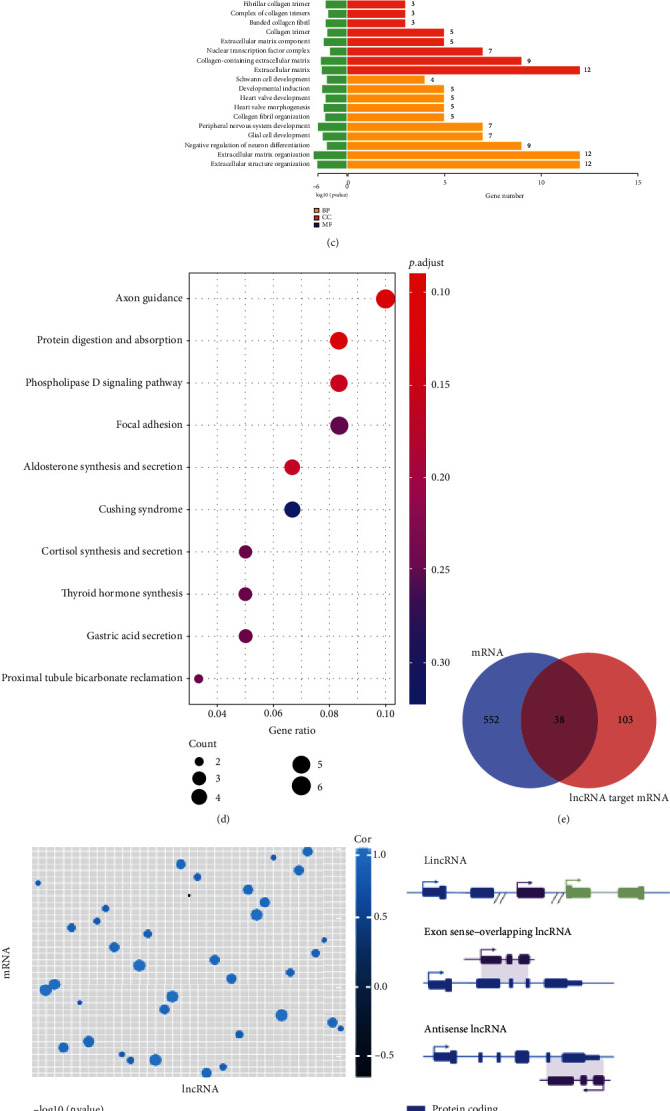
Difference analysis of lncRNA. (a) Heatmap of DE-lncRNAs (top 50). (b) Volcano figure includes only data with a *p* value < 0.05. (c) Every ten most significantly enriched GO term was selected from the three categories of BP (purple), CC (orange), and MF (yellow) and is displayed in the figure. If there were less than 10 GO terms, they were all displayed. (d) Top 10 KEGG pathways. (e) Venn diagram of the intersection of DEGs and DE-lncRNA target mRNA. (f) Scatter diagram of the correlation between DEGs and DE-lncRNAs. If the correlation is greater, the color is lighter; if the point is bigger, the *p* value is smaller. (g) Diagrammatic drawing of lncRNAs which were matched to mRNAs. Arrows represent the transcription directions, and double slashes separate the two sequences that encode the protein. (h) lncRNA-mRNA cis regulatory interaction network diagram. The picture shows interactions with correlation *p* value < 0.05 only.

During tissue differentiation and development, most lncRNAs have obvious spatiotemporal expression specificity. Therefore, we wanted to investigate their influence on development. lncRNA regulates the expression of corresponding genes by binding to the target mRNA, and minute changes in lncRNA expression will alter gene expression. Thus, firstly, we explored the targeting relationship between mRNA and DE-lncRNA by lncTar software. By calculating the minimum free energy (ndG) of lncRNA and mRNA binding sites, it was determined whether they can generate stable binding sites. A total of 369 lncRNAs were upregulated, and 182 were downregulated from the bell differentiation stage to the secretory stage of the rat molar. In total, 165 DE-lncRNAs were predicted to target genes (Figures 3(a) and 3(b)). The differential target lncRNAs (DTL) were also classified by GO categories which included 407 terms significantly related to BP, 28 to CC, and 38 to MF (Figure 3(c)). We noticed that lncRNA mainly combined with enhancer and proximal promoter DNA and also participated in transcription activity. Furthermore, lncRNAs were also involved in ECM remodeling which was based on sodium, a special alternative to collagen. They, in detail, were closely related to constituents of fibrillar collagen trimer (GO:0005583), complex of collagen trimers (GO:0098644), and banded collagen fibril (GO:0098643). In our result of GO enrichment, we found that odontogenesis of dentin-containing tooth (GO:0042475), regulation of tooth mineralization (GO:0070170), regulation of odontogenesis of dentin-containing tooth (GO:0042487), and tooth mineralization (GO:0034505) were also relative to the formation of hard tissue in teeth. Additionally, some lncRNAs linked to Schwann cells (GO:0014044), heart valve (GO:0003170), peripheral nervous system (GO:0007422), glial cell (GO:0021782), and other organ development were also identified. Furthermore, KEGG pathway annotation showed that DE-lncRNAs were enriched in protein digestion and absorption (rno04974), axon guidance (rno04360), phospholipase D signaling pathway (rno04072), aldosterone synthesis and secretion (rno04925), and ultimately, 15 pathways (Figure 3(d)).

To identify the coexpression between mRNA and lncRNA, we made the intersection of lncRNA target mRNA and DEGs and revealed that 38 genes were coexpressed (Figure 3(e)). The cor.test function of the R package was used to clarify the correlation analysis (Figure 3(f)). Based on the differential genes and target prediction, the cis regulatory relationship showed that there were 37 DE-lncRNA coexpressed with the 36 DEGs including 36 genes with accordant tendencies (Figure 3(h), Table 3), containing 27 pairs of synchronous upregulated, 9 pairs of synchronous downregulated, and 1 pair of lncRNA upregulated-mRNA downregulated data. In contrast to the NONCODE database (http://www.noncode.org/) which is a comprehensive database of noncoding RNAs [31], the types of coexpressive lncRNA were mainly long intergenic noncoding RNA (lincRNA), exon sense-overlapping lncRNA, and antisense lncRNA (Figure 3(g)). Just as the name implies, lincRNA is located between genes and usually found within <10 kb of protein-coding genes in mammalian cells and is important in embryonic development [32]. It is demonstrated that targets of lincRNA can be divided into cis and trans ones. The cis target is found only in proximity to the lincRNA gene. The potential mechanism relies on transcription or the nascent RNA or depends on processed RNA as well as the site of transcription. The trans one can be found anywhere in cells and has nothing to do with transcription sites [33]. As a member of the lncRNA category, the antisense lncRNA is also deemed a transcriptional product of RNA polymerase II and can increase the risk of illness. ANRIL, for instance, an antisense lncRNA encoded at CDKN2A/B genomic locus, regulates gene expression by chromatin regulation of transcription factor binding and miRNA regulation [34]. Nevertheless, there are few data about the function of exon sense-overlapping lncRNA, which need to be studied. To summarize, no matter what kinds of lncRNA, they all perform a function in cis and take part in the regulation of neighboring protein-coding machinery.

### 3.3. DNA Methylation Influenced mRNA

**Figure 4:**
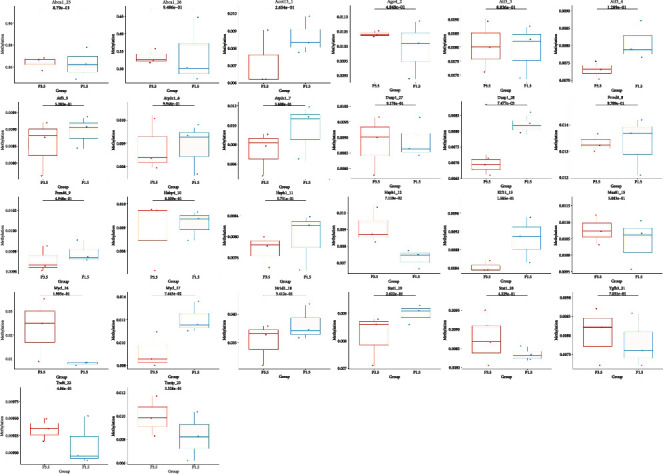
Boxplot of fragment mean methylation level. The *X*-axis was the two groups for the difference analysis; the *Y*-axis was the average methylation level of each target fragment in the group. The box represented the overall average methylation level of all samples in the group on the target fragment, and the point represented the average methylation level of each sample on the target fragment.

To identify vital genes, we took into consideration genes with important cell functions such as cell growth and proliferation, cell cycle regulation, cell stress response, and DNA replication and repair. Ultimately, 32 fragments with 31 methylation sites of 18 genes from our transcriptome data were chosen for MethylTarget targeted bisulfite sequencing. These genes included the following: *Abca1*, *Acot13*, *Ago4*, *Atf3*, *Atp2c1*, *Cklf*, *Dusp1*, *Frmd6*, *Habp4*, *Hsps1*, *Klf11*, *Msu8 1*, *Mycl*, *Nr1d1*, *Stat1*, *Tgfb3*, *Traf6*, and *Txnip*. All detected fragments had no methylation difference at the gene level, but regarding site, target, and haplotype, there was differential expression. In the process of tooth germ development, the change of methylated CpG sites referred to chromosome (chr) 2 (*Txnip*_23, located in 75^th^ position), chr8 (*Atp2c1*_7, located in 244^th^ position), chr10 (*Dusp1*_28, located in 217^th^ position), chr5 (*Ago4*_2, located in 204^th^ position), and chr17 (*Acot13*_1, located in 22^nd^ position); 26 fragment targets had differential expression in the mean methylation level (Figure 4). Moreover, the haplotype of *Habp4_10* and *Dusp1_28* significantly changed, but only *Dusp1_28* showed different methylation levels. The mean methylation level of *Dusp1_28* was significantly decreased (*p* = 0.007). Regional methylation in the promoter affects mRNA transcription levels, and there are negative correlations between levels of DNA methylation and gene expression [35]. It explained that the mRNA expression level of *Dusp1* was incresed in the result of RT-qPCR..

### 3.4. Common Influence of lncRNA and DNA Methylation on mRNA

**Figure 5:**
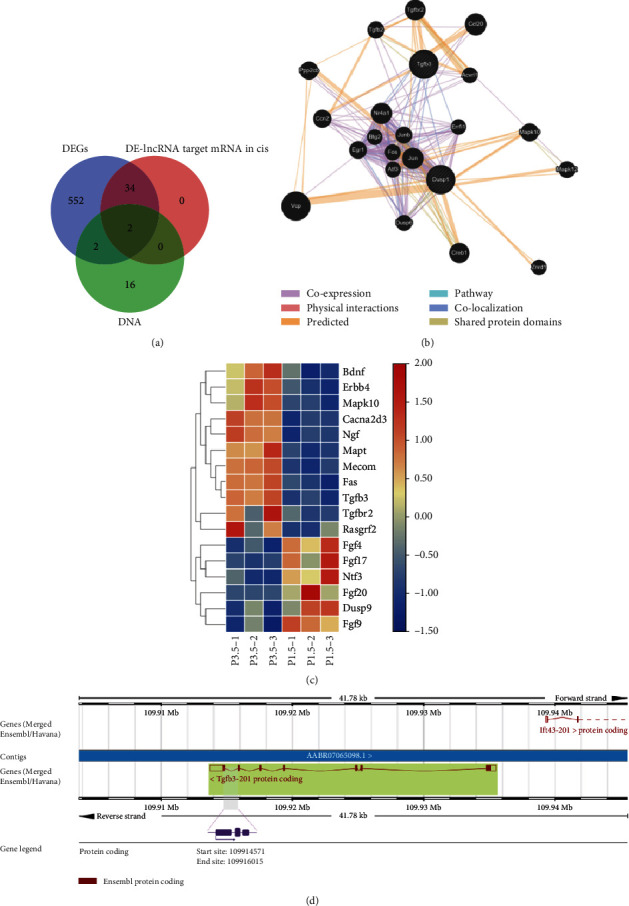
Interaction between *Tgfb3* and *Dusp1*. (a) Intersection among DEGs and DE-lncRNA target genes of cis regulatory and selected methylation test DNA. (b) GeneMANIA network identifying potential interactions with *Tgfb3* and *Dusp1* associated with MAPK signaling pathway in *Rattus norvegicus*. (c) Heatmap of MAPK signaling pathway map. (d) *Tgfb3* gene was in a reverse strand of chromosome 6 and started from 109,913,757 to 109,935,533. The purple region was *Tgfb3* lncRNA which is located from 109,914,571 to 109,916,015.

We wanted to find out if lncRNA can influence methylation or if together they influence mRNA expression in some way. We made a Venn diagram to overlap DEGs and DE-lncRNAs and tested DNA and found two overlapping genes, *Nr1d1* and *Tgfb3* (Figure 5(a)). *Nr1d1* mainly took part in response to molecules of bacterial origin (GO:0002237), response to lipopolysaccharide (GO:0032496), regulation of protein secretion (GO:0050708), epithelial cell proliferation (GO:0050673), regulation of epithelial cell proliferation (GO:0050678), and other biological processes. Thereunto, the effect of *Nr1d1* on epithelial cell proliferation is important for tooth germ development but needs further research. Moreover, in KEGG enrichment analyses, *Nr1d1* was enriched in circadian rhythm (rno04710). *Tgfb3* was involved in most biological processes and KEGG pathway, for example, ECM (GO:0031012), collagen-containing ECM (GO:0062023), receptor regulator activity (GO:0030545), biomineral tissue development (GO:0031214), cytokine-cytokine receptor interaction (rno04060), and MAPK signaling pathway (rno04010). However, the target fragment we detected of those two genes had no change in DNA methylation level, site, target, or haplotype.

According to our results, *Tgfb3* was significantly upregulated at both the mRNA and lncRNA expression levels, and it may be caused by *Tgfb3* lncRNA overlap with its protein coding sequence from the sixth to seventh exons and regulated mRNA in cis (Figure 5(d)). Interestingly, its DNA methylation status had barely changed, which demonstrated that lncRNA may restrain the methylation level. Furthermore, we found a potential relationship between genes *Tgfb3* and *Dusp1*, considering they were all one member of the MAPK signaling pathway (data not shown). In parallel, we used GeneMANIA (http://genemania.org) to further verify whether there was really a relationship between the two. GeneMANIA is reported as a website for generating hypotheses about gene function, analyzing gene lists, and prioritizing genes for functional assays [36]. According to GeneMANIA, there were 20 genes related to the connection of *Tgfb3* and *Dusp1*. Among those genes, *Tgfb3* coexpressed with *Jun*, *Nr4a1*, and *Ccn2* and colocalized with *Dusp1*, *Errfi1*, *Egr1*, *Jun*, *Junb*, *Fos*, *Atf3*, and *Nr4a1*, while Dusp1 coexpressed with Dusp6, *Ccn2*, *Egr1*, *Btg2*, *Fos*, *Atf3*, *Jun*, *Junb*, *Nr4a1*, *Errfi1*, and *Tgfb2* and colocalized with *Dusp6*, *Egr1*, *Fos*, *Junb*, *Nr4a1*, and *Tgfb3* (Figure 5(b)). Although these data suggest a relationship, whether their common localization is because they are expressed in the same tissue or their gene products are identified in the same cell location is still uncertain. In addition, according to KEGG analysis, *Tgfb3* and *Dusp1* are both involved in the MAPK signaling pathway. Besides, as mentioned earlier, CD147 modulates MMP20 through the MAPK signaling pathway. Indeed, the MAPK pathway was significantly altered in the late bell stage. Therefore, we analyzed DE-mRNA and constructed a heatmap of this signaling pathway (Figure 5(c)).

### 3.5. The Result of RT-qPCR

**Figure 6:**
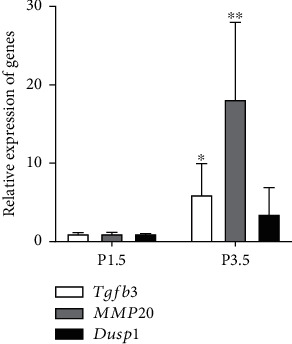
Relative expression of *Tgfb3*, *MMP20*, and *Dusp1*. (^∗^Compared to the 1.5^th^ group, *p* < 0.05; ^∗∗^compared to the 1.5^th^ group, *p* < 0.01.) The column height indicates the average degree of genes' relative expression. The short bar at the top of the column indicates the standard deviation of the degree of genes' relative expression.

In the aforementioned data analysis, we found that the MAPK signaling pathway played an important role in the process from the period of the bell differentiation stage to the secretory stage. And we noticed that the expression of *Tgfb3*, *MMP20*, and *Dusp1* was closely related to this pathway. To confirm the reliability of its expression trend, we performed RT-qPCR validation. In our validation experiments, we found that the expression levels of *Tgfb3*, *MMP20*, and *Dusp1* increased during development (Figure 6). Among them, the expression of *Tgfb3* and *MMP20* in molar tooth germ in the group at 3.5^th^ days after birth was significantly higher than that in the group at 1.5^th^ days after birth (*p* < 0.05). However, the expression of *Dusp1* has no statistically significant difference. This result was consistent with our RNA sequencing results. However, due to individual differences, gene expression levels varied, resulting in large standard deviations. But the overall trend was the same.

## 4. Discussion

Many studies have explored the genes involved in tooth germ development and revealed that the Wnt, BMP, TGF-beta, Shh, and MAPK signaling pathways are involved [37]. However, few studies associate transcriptomics with epigenetics. From the bell differentiation stage to the secretory stage, the enamel matrix and dentin matrix are secreted and the tooth ECM undergoes vast remodeling. To clarify what is influenced during this period, we not only sequenced the transcriptome level of its mRNA but also combined lncRNA and DNA methylation levels to conduct a systematic characterization of the late bell stage of tooth germ development.

There is no doubt that the regulation of gene expression during tooth embryo development is a complex network regulation. In the study, we revealed multiple signal changes with a total of 590 DEGs with most of them being enriched in odontogenesis, amelogenesis, and ECM remodeling-related terms with odontogenesis (GO:0042476), ECM (GO:0031012), collagen-containing ECM (GO:0062023), odontogenesis of dentin-containing tooth (GO:0042475), ECM structural constituent (GO:0005201), and amelogenesis (GO:0097186). While KEGG enrichment results revealed that CAMs (rno04514), ECM-receptor interaction (rno04512), cytokine-cytokine receptor interaction (rno04060), PI3K-Akt signaling pathway (rno04151), and MAPK signaling pathway (rno04010) were related to tooth development. In reviewing the results, we noticed that *MMP20* gene expression not only influenced ECM remodeling but also was related to sodium ion transferred through its solute carrier Slc24a4. However, the latter mechanism needs further study. We also observed that 37 DE-lncRNAs influenced mRNA expression by cis regulation, and lncRNA may affect DNA methylation. Combining 32 target fragments of DNA methylation results, we finally obtained three genes, *Nr1d1*, *Tgfb3*, and *Dusp1*. Although *Nr1d1* is the exception (which was involved in epithelial cell proliferation), both *Tgfb3* and *Dusp1*, the same as *MMP20*, associate with the MAPK signaling pathway.

The MAPK pathway has shown to be involved in the development of eukaryotes such as plants and vertebrates [38, 39] by regulating cellular proliferation and differentiation. Along with tooth development, odontoblastic differentiation was influenced by the MAPK signaling pathway, especially p38 MAPK and Erk 1/2 signaling [40]. In our study, we found out that MAPK plays a vital role during the transformation from the bell differentiation stage to the secretory stage. Its effect was not restricted to gene expression but extended to epigenetic regulation. On the one hand, CD147 upregulates MMP20 through the MAPK pathway, and then, MMP20 degrades collagen, elastin, and DSPP to remodel the ECM. Changes in the ECM microenvironment can induce stem cell differentiation. On the other hand, as well as *MPP20*, *Tgfb3* contributes to cellular component disassembly and ECM cellular component. Coincidentally, *Tgfb3* is also involved in regulating the MAPK pathway [41]. We found that *Tgfb3* colocalized with *Dusp1* which is known as a crucial participator in multiple diseases and cancers [42]. Its hypermethylation in promoter regions of genes can induce oral squamous cell carcinoma [43]. In fact, DUSP1 is a regulator of cell growth and survival in both physiological and pathological processes. The mechanism suggests that *Dusp1* negatively regulates MAPK by phosphorylation or dephosphorylation of the activated protein and then influences the downstream cellular reaction of the MAPK signaling pathway [44, 45]. In our DNA methylation test, *Dusp1* maintained hypomethylation by change of site, target, and haplotype after going through the second stage of demethylation. DNA methylation has been reported to suppress transcription through direct or indirect mechanisms. When methylation occurs in the promoter region, the CpG sequence of the transcription binding site changes and contributes to a decrease in the ability of transcription factors bound to sites, which directly affects the transcription level of genes [46]. Furthermore, DNA methylation can alter the chromatin state or recruit chromatin remodeler to influence the binding between transcription and DNA indirectly [47]. In summary, there is an inverse correlation between DNA methylation levels and gene expression. Thus, in the fragment area of *Dusp1_28*, the regional methylation was decreased, and the mRNA transcription was increased slightly. Under the influence of *Dusp1*, the MAPK signaling pathway was activated and statistically enriched to enhance cellular quantity and dentin or enamel differentiation.

We sequenced the whole tooth germ tissue and revealed the gene regulation network from bell stage differentiation to the secretion stage of rat tooth embryo development. The MAPK pathway was particularly valuable to tooth development. Associated with this signaling pathway, *MMP20* and *Tgfb3* were upregulated, while *Dusp1* had no significant difference at the mRNA level which was confirmed by RT-qPCR. Interestingly, the target DNA fragment methylation of *Tgfb3* and the mRNA expression of *Dusp1* had no statistical difference. The reason may be that our experiment did not separate epithelial and mesenchymal tissue, and the offset of expression level may cover up some variables. Separation of tissue components is a better way to comprehensively reflect whole genome methylation and identify differential information.

## 5. Conclusion

In the current study, we combined transcriptomics with epigenetics to explore the mechanism underlying the development of mandibular molars and revealed a gene regulation network from bell stage differentiation to the secretion stage of rat tooth germ development. We noted that during this period, along with secretions of enamel and dentin matrix and the occurrence of hard tissues, the ECM was remodeled via the MAPK signaling pathway. Sodium ion and metal ion channels were activated, collagens were degraded, and DSPP was increased. All the aforementioned alterations were affected by *MMP20*, *Dusp1*, and *Tgfb3*; however, the molecular mechanism is still unclear, and further research is required.

## Figures and Tables

**Table 1:** DNA methylation primers.

Genes	Primers (5′⟶3′)	Length (bp)
Abca1	F: GTTGGAATAGGGATAGAAGTTTTTAAAG R: CAACACAAAATAAAAACTCATAAATCATC	282
Abca1	F: GAGTTTTTGTAATATTATTTGTATTGTGATTAG R: TTATTCCAAAAACCTACTCATACTAAAACAC	281
Acot13	F: TGTYGGAGGTTGGTGAGGT R: AACTACRACACTACRATAACTTCCACTTT	202
Ago4	F: TAGTGTTYGGTTYGGGGATAGG R: CCCCCTACCTCRAACCTACTC	237
Atf3	F: TTYGYGTTTYGGTAGAGTTTTTGG R: AATCCCCAATAACACAAAAATCC	194
Atf3	F: AGGATYGYGATTTTTAGAGTGTGG R: CCRAACCCTTATACCACTAAAAACC	262
Atf3	F: AATAGYGAATGAGGTTGGAAGTGA R: AAAATCCRCACAACCRCAAAAA	220
Atp2c1	F: GGGGGAAYGAGATYGAGATAGG R: CAAAACCAAAAACRCAACCAAAA	207
Atp2c1	F: TTTTGAGAGTTTAAGGGTTAGTTTTG R: TCCCRCCTCCACCTCCTAC	263
Dusp1	F: GGGTAGGGGAGTAGGGTAGGT R: CCACCAAAACCAAAAACAAAAAC	186
Dusp1	F: GGTGGGTGGGTGATTTGTTT R: TTCTACCACCTCCCAACCTCTC	268
Frmd6	F: TTATTTTAATTTGGGGAGATAGATTGAG R: TCTACACACCCTAATCCCCTAAC	268
Frmd6	F: GGGAGGTYGGGGTTTTTAGG R: ACACAACAAACAATACCRCACATC	186
Habp4	F: GAGTGGGAGGGATAAAGTGG R: CCCAAAACCCCCTTCATAAC	255
Hsph1	F: GAGAGTATGTYGGGAAGYGTAGTTTG R: AACAAAACACAAAACAAAACATACACC	228
Hsph1	F: GGGGYGAGTTTGGAAGGTTAG R: CCAAACAAAAACRAACCRAACC	263
Klf11	F: YGATTGGTTGGYGGTAGAGG R: CACCTCCRCCCTCCTTACC	159
Mus81	F: YGGAAATATGGTGAGTATTAGGTTATTG R: CCTACCCTTCAATAAAACCCTTAATC	156
Mycl	F: GGGTTGTAAGGTTGTAGATTGTTTT R: AAACTCTAATCCCTCCCCAAAC	233
Mycl	F: TYGTGGTTAGGAGGGGGTGT R: CCCCCRAAACACTCCTAAAA	174
Nr1d1	F: TTTTGGGATAGAGGGTTTTGYGTAG R: TCAATAAACTACAAATCCCAACAATC	189
Stat1	F: GTTGGGATTGGTYGGTTGTT R: TTCTCCAACCRTATAAACAAAACCTC	164
Stat1	F: AGAGGATGGAATAGTGTTGATATGATTG R: AAACTAAAATCTACTACCCACAAATAACCT	259
Tgfb3	F: TAGGTTAGGTTYGTTAGGGGAGA R: AATACCATCATTTACCCCAACAAC	150
Traf6	F: GGTTTTTGGGTYGYGGTTATTG R: AAAAACCCTCTCTCCCRTAAAA	153
Txnip	F: TGGATATTTAAAGATTAGAAGAGGAGAGTG R: CCCCTCCTCCCTAACAAAAC	261

**Table 2:** RT-qPCR primers.

Genes	Primers (5′⟶3′)	Length (bp)
*Tgfb3*	F: CTCCCCCTTTCTACTGGGGT R: AAGTCCAAGGTGGTGCAAGT	167
*MMP20*	F: TTGCTGCTCACGAATTTGGC R: TCCTTGGGGAGATGGAACCT	115
*Dusp1*	F: GCTCATGTGAGCTGGTCCTT R: TGTCAGAGGGGCTCGATGTA	165

**Table 3:** lncRNA and its target mRNA in cis.

lncRNA	Location	lncRNA_regulation	Target mRNA	mRNA_regulation	Correlation
NONRATG008597.2	Linc	Up	Egr3	Up	1
NONRATG001431.2	Exonic	Down	Golga7b	Down	1
NONRATG007993.2	Exonic	Up	Ambn	Up	1
NONRATG001139.2	Exonic	Down	Fgf4	Down	1
NONRATG018442.2	Exonic	Up	Col9a2	Up	1
NONRATG015154.2	Exonic	Down	Prom2	Down	1
NONRATG007649.2	Linc	Down	LOC310926	Down	1
NONRATG003202.2	Exonic	Up	Bmerb1	Up	0.9411
NONRATG011008.2	Antisense	Up	Ablim3	Up	0.9428
NONRATG013344.2	Antisense	Down	Sox2	Down	0.9428
NONRATG012390.2	Linc	Up	Hey1	Up	0.9428
NONRATG008875.2	Exonic	Up	Tgm1	Up	0.9428
NONRATG008146.2	Exonic	Up	Crmp1	Up	0.9428
NONRATG020673.2	Exonic	Up	Tnfaip2	Up	0.9428
NONRATG004794.2	Exonic	Up	Nr1d1	Up	0.9428
NONRATG001943.2	Exonic	Up	Bcl3	Up	0.9428
NONRATG023047.2	Exonic	Up	Col5a3	Up	0.9428
NONRATG015793.2	Linc	Up	Slc38a11	Up	0.9428
NONRATG020568.2	Exonic	Up	Tgfb3	Up	0.9428
NONRATG011271.2	Exonic	Up	Lox	Up	0.9428
NONRATG007763.2	Exonic	Up	Pik3ip1	Up	0.8857
NONRATG002964.2	Exonic	Up	Pip5k1b	Up	0.8857
NONRATG019669.2	Antisense	Up	Kcnk3	Up	0.8857
NONRATG021992.2	Exonic	Down	Ly6h	Down	0.8857
NONRATG013084.2	Exonic	Down	Cartpt	Down	0.8261
NONRATG007559.2	Antisense	Up	Ambn	Up	0.8285
NONRATG010089.2	Exonic	Down	Tfap2a	Down	0.8285
NONRATG003939.2	Exonic	Up	Rundc3a	Up	0.8285
NONRATG003513.2	Exonic	Up	Pmp22	Up	0.8285
NONRATG012335.2	Antisense	Up	Adamts12	Up	0.8285
NONRATG000639.2	Antisense	Up	Synm	Up	0.7714
NONRATG024442.2	Exonic	Up	Col5a2	Up	0.7714
NONRATG007316.2	Exonic	Up	Atp1a2	Up	0.7714
NONRATG022623.2	Exonic	Down	Pclaf	Down	0.7714
NONRATG017361.2	Exonic	Up	Tmem229a	Up	0.7714
NONRATG003057.2	Exonic	Up	Crtac1	Up	0.6982
NONRATG009280.2	Exonic	Down	Slc5a5	Up	-0.6571

Note: the table is sorted in order of relevance.

## Data Availability

The data used to support the findings of this study are available from the corresponding authors upon request.
